# Gastric Volvulus: A Multidisciplinary Approach and Conservative Treatment

**DOI:** 10.7759/cureus.13285

**Published:** 2021-02-11

**Authors:** Carina Rodrigues, Isabel Taveira, Ana Deus, Henrique Rita

**Affiliations:** 1 Unidade de Cuidados Personalizados de Sines Family Medicine, Hospital Do Litoral Alentejano, Santiago do Cacém, PRT; 2 Internal Medicine, Hospital Do Litoral Alentejano, Santiago do Cacém, PRT; 3 General Surgery, Hospital Do Litoral Alentejano, Santiago do Cacém, PRT

**Keywords:** gastric volvulus, conservative treatment, multidisciplinary, chronic disease

## Abstract

Gastric volvulus can be defined as an abnormal rotation of the stomach. It can be both an emergency and a chronic intermittent problem. Being such a rare clinical entity and a difficult condition to diagnose, it is commonly diagnosed at the time of surgery or even at autopsy.

We present the case of an 82-year-old independent female with a past medical history of hiatal hernia, who came to the emergency department with hematemesis and severe epigastric pain. An abdominal CT scan revealed an organoaxial stomach volvulus within the intrathoracic cavity.

After initial treatment with gastric decompression, IV fluids and proton pump inhibitors, the patient was informed that the surgical intervention would be the only definitive curative treatment for her condition and denied the procedure. She was then discharged after clinical and analytical improvement. She was offered a conservative treatment and follow up by the internal medicine team. After a few months, the patient reported moderate improvement of her symptoms and less episodes of epigastric pain. She was pleased with the conservative management and denied any surgical or invasive procedures. A proximity contact was established with the family doctor, which she maintains.

This case report is proof that rare entities can happen to patients presenting common symptoms and better resolutions come from multidisciplinary approaches.

## Introduction

The first report of gastric volvulus dates from 1579, by Paré, who described a patient with a diaphragmatic injury due to sword, but it was only in 1886 that Berti described this entity for the first time. Eighteen years later, Borchardt reported the Gastric volvulus triad which consisted in acute stomach distention or pain, inability to pass a nasogastric tube and non-productive attempts at vomiting [1,2]. Since then, with all the following scientific, medical and technological developments, this entity been thoroughly described and classified within the clinical, diagnostic and therapeutical elements [1,2].

Gastric volvulus can be categorized as acute or chronic, primary or secondary (regarding etiology) or even based upon the axis of abnormal rotation (long versus short axis). There are different diagnostic methods, each one with different advantages. Gastric volvulus is usually approached surgically, however, there are other approaches like the medical and conservative treatment to selected patients. Although is it a well-known entity, because of its infrequency, it is rarely suspected and many diagnoses are missed until surgery or even autopsy [2,3].

## Case presentation

An 82-year-old female, previously independent, presents to the emergency department, complaining of worsening epigastric pain, eructation, heartburn, nausea and vomiting for the last two days. She described the pain as a burning sensation, cyclic and severe in nature. The pain irradiated to her mouth and to the left hypochondria. The patient did not identify triggering, relieve or aggravating factors. She had multiple food contenting vomiting episodes with an occasional small amount of bright blood. She also complained about progressive anorexia, fatigue and asthenia, with increasing weight lost, in the last year. She denied any other complaints, either gastrointestinal or systemic. She already had similar complaints in the past one and half years that motivated previous multiple emergency episodes where she was diagnosed with gastroesophageal reflux disease and medicated accordingly.

Past medical history was significant for hypertension, angina pectoris, osteoporosis, osteoarthrosis and hiatal hernia. In the previous year, she had denied surgical intervention to repair the hiatus hernia. Her usual medications included amitriptyline, brotizolam, alprazolam, nicorandil, gabapentin, valsartan, omeprazole, and cholecalciferol. Physical examination was notable only for dry skin and mucosa, and generally painful abdominal palpation with a major compromise of the epigastric and left hypochondria. Laboratory studies were pertinent for peripheral white blood cells of 13.2 × 10^3^/µL with elevated neutrophil count of 87% and lymphocytopenia of 7.6%. Lactate dehydrogenase (LDH) was 896 UI/L, amylase was 110 U/L and ultrasensitive troponin was 987 ng/ml.

**Figure 1:**
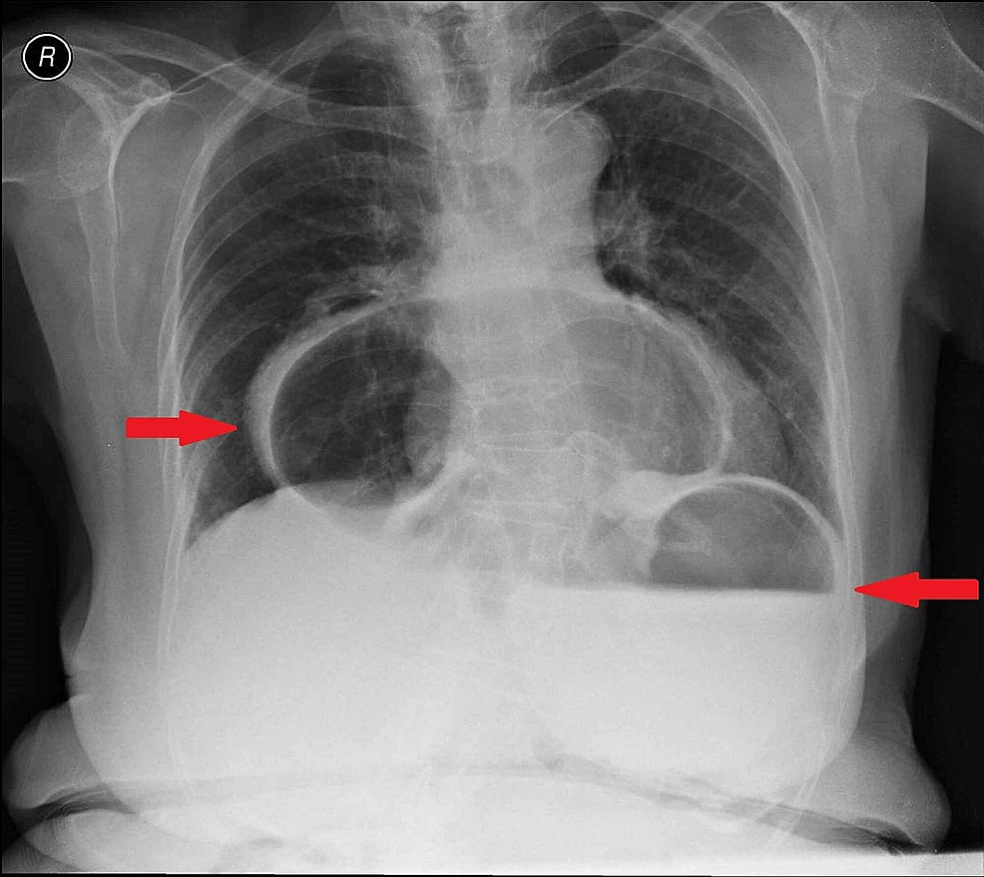
Chest radiography showing wide hernia of the hiatus June, 2016. The right arrow shows a stomach portion within the thoracic cavity and the left arrow shows another stomach portion within the abdominal cavity.

**Figure 2:**
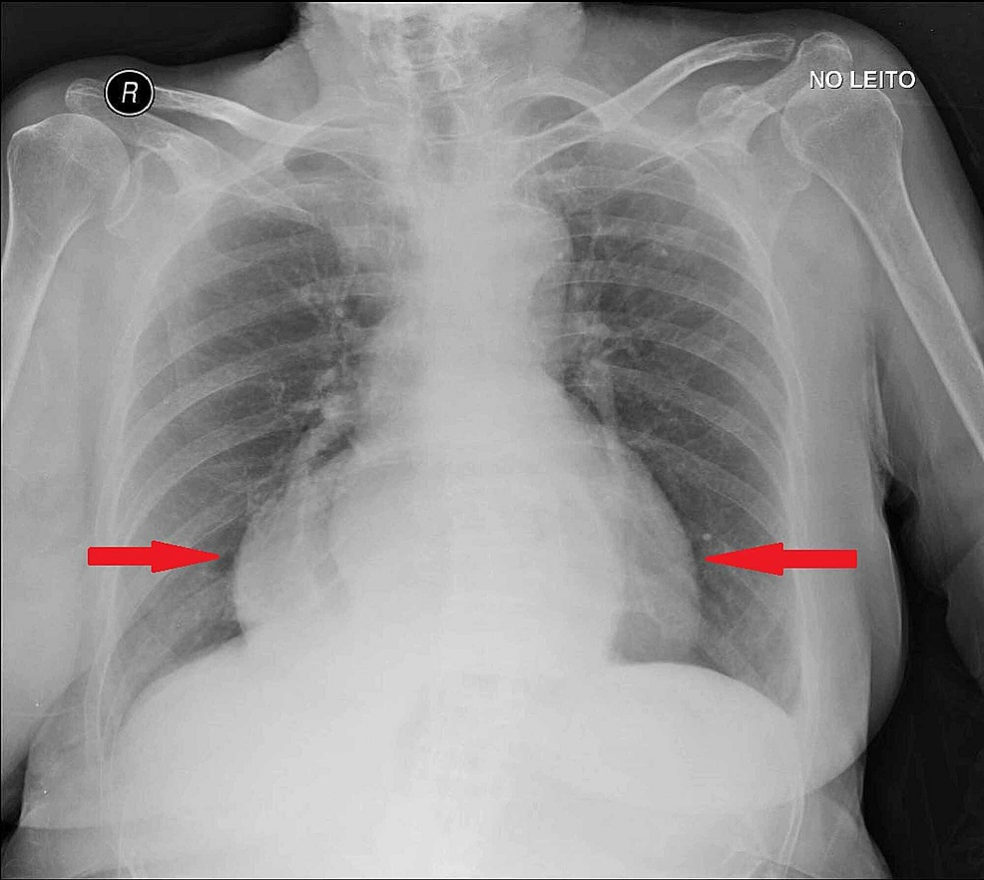
Chest radiography showing wide hernia of the hiatus May, 2020. The arrows show the stomach within the thoracic cavity.

An electrocardiogram was performed that showed no alterations. A chest x-ray was also performed and compared to a previous from 2016. As you can see in the two pictures below (Figures 1 and 2), both X-rays revealed a significantly large hiatus hernia with a portion of stomach within the intrathoracic cavity.

**Figure 3:**
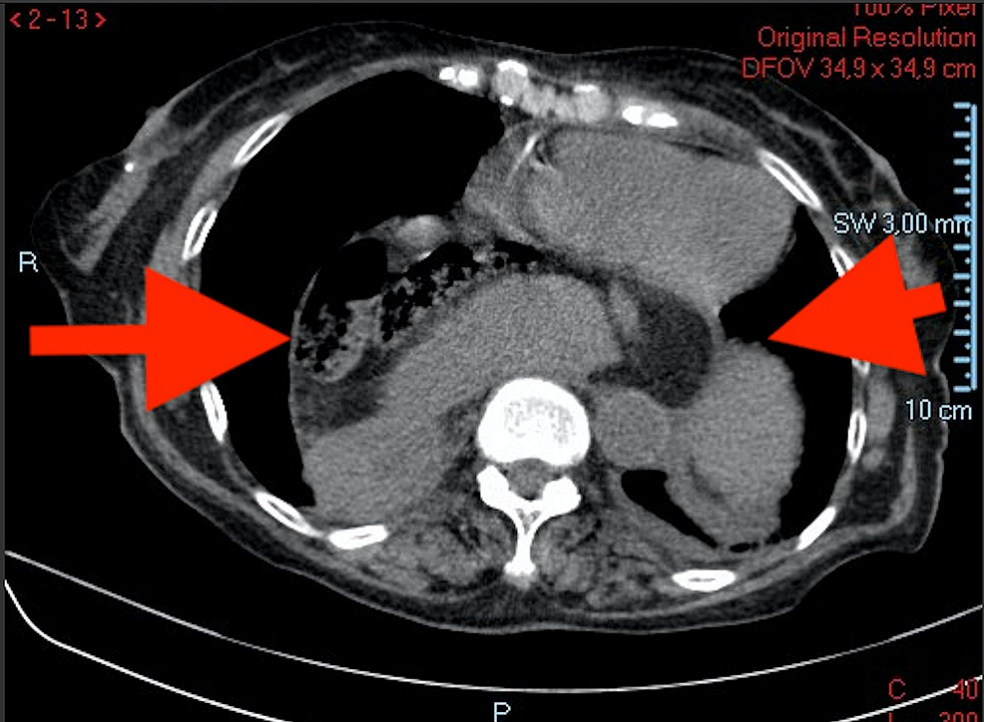
Transverse segment of a CT scan showing a distended stomach, with a suggestion of gastric volvulus in the intrathoracic segment. The arrows show the stomach within the thoracic cavity.

**Figure 4:**
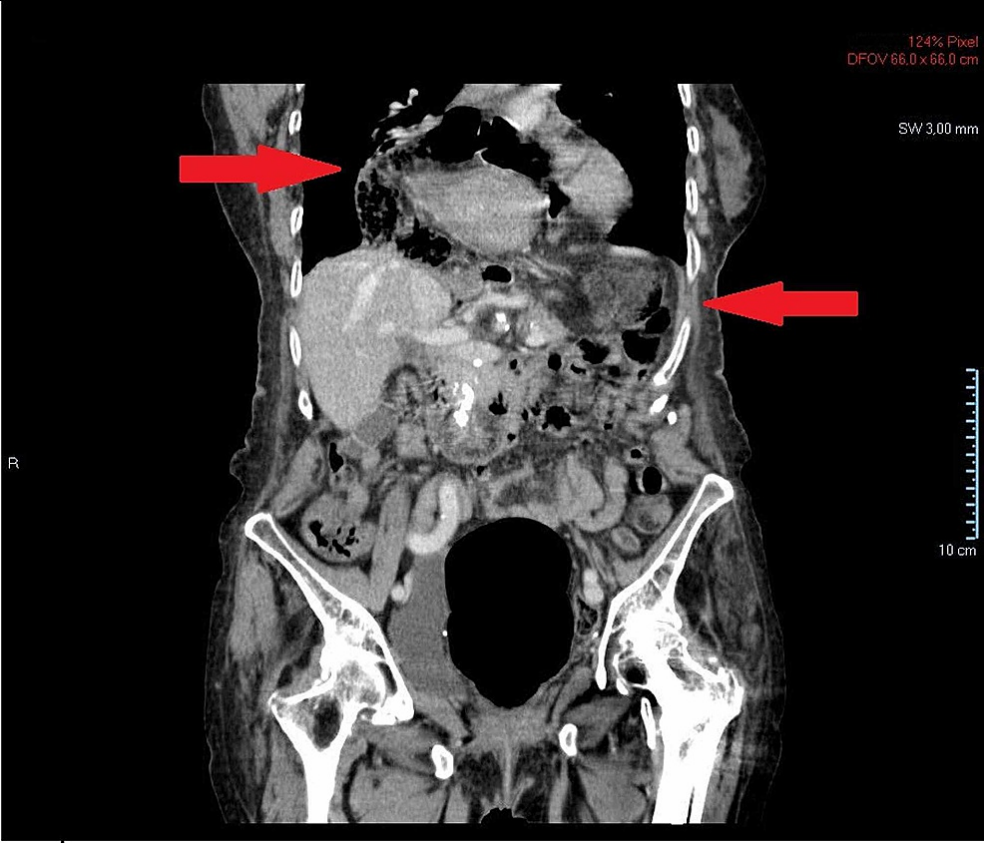
Coronal segment of a CT scan showing a distended stomach, with a major portion in the intrathoracic segment. The right arrow shows a stomach portion within the thoracic cavity and the left arrow shows another stomach portion within the abdominal cavity.

A contrast abdominal and pelvic CT scan allowed the visualization of a very large herniation of the oesophageal hiatus with herniation of a large part of the stomach to the intrathoracic topography, as depicted in the two pictures below (Figures 3 and 4). The stomach was very distended, with a suggestion of gastric volvulus in the intrathoracic segment, suggestive of a type 1 gastric volvulus (organoaxial rotation). No other significant alterations were found, including focal thickening of the gastric wall.

The case was approached by Internal Medicine and General Surgery, and she was admitted to the Observation department. After clinical and radiological evaluation, it was assumed that there was no apparent wall ischemia or perforation and that there was indeed stomach distention in both intrathoracic and intraperitoneal portions. The volvulus appeared to be the organoaxial type.

The patient was treated with nasogastric decompression, after a difficult nasogastric intubation, zero diet, fluid therapy, a prokinetic and a proton pump inhibitor. In the following day, the patient started a clear liquid diet which she tolerated without complications, allowing the progression to solid aliments afterwards. After two days under observation, since she presented a clear clinical improvement, she was discharged being advised to perform an upper endoscopy.

Attempts to schedule an internal medicine consultation were made unsuccessfully due to the patient’s scarcity of social support. This factor originated a few more urgency department episodes, with similar but yet milder symptoms the did not require admission. An approach by phone was sought. Once again, treatment possibilities and clinical symptoms were explained. The patient was fully aware that the conservative treatment would not prevent the recurrence of episodes but might control the symptoms. Clear communication was attempted, and the team was explicit about supporting and respecting either of the treatment decisions. Due to her age and multiple comorbidities, the patient decided to decline surgery and any invasive intervention including the suggested upper gastrointestinal endoscopy, even though she was aware that this examination could have both diagnosis and treatment value.

Finally, a solid link with the family doctor was made, which has been very beneficial for the patient's physical and mental well-being. Feeling more supported, her anxiety level is lower, and the emergency department episodes became scarcer in the following months. In the last follow up call, the patient reported improvement under dietary advice and pharmacological treatment directed at her symptoms.

It was explained to the patient that she was entitled to change her decision about the treatment choice at any time and that the hospital team would be available to support her if she does. Although no clinical resolution of the problem has been achieved, a balance has been accomplished through multidisciplinary effort, leading to the optimal possible resolution. 

## Discussion

Gastric volvulus is an abnormal stomach rotation that ordinarily arises in association with a large hiatal hernia. Up to 2/3 of the volvulus occur above the diaphragm. The incidence is not completely known but it is believed that it peaks after the fifth decade with adults comprising 80-90% of cases. The remainder of cases occurs in 15-20% of children below one-year-old, especially when associated with congenital diaphragm abnormality. It affects both sexes in the same way. Other associated risk factors are diaphragmatic abnormalities like paraesophageal hernia or hiatal hernia, diaphragmatic eventration, phrenic nerve paralysis, kyphoscoliosis, focal adhesions, gastric tumors or other gastrointestinal abnormalities [1-3].

There are many possible categorizations of gastric volvulus, such as by etiology, type of rotation or clinical presentation. Etiologically, if it is due to gastric ligament abnormalities, it is considered primary or idiopathic, but this presentation represents only one-third of the cases. When other anatomic abnormalities, like a paraoesophageal or hiatal hernia, are the cause, it is designated secondary [3]. There are four subgroups of gastric volvulus according to the axis of rotation. The stomach can be twisted in its long axis from the esophagogastric junction to the pylorus being described as organoaxial, type I, comprising approximately 60% of cases. This type may lead to obstruction at the gastroesophageal junction, at the pylorus, or both. Generally, it presents as an acute event. The stomach can be twisted along its short axis, mesenteroaxial rotation, type II, where the most important risk factor is the laxity of the gastrosplenic ligament. Usually, the volvulus is intermittent, incomplete, and chronic. Type III rotation is a combined form of I and II and is the least common representing only 2% of cases. Type IV is “unclassified” and accounts for approximately 10% of cases [4].

The patient presentation can vary from an acute and very symptomatic to a chronic condition that is often asymptomatic. There can be a complete gastric outlet obstruction or a partial one, presenting with an intermittent presentation. Borchardt's triad occurs in as many as 70% of acute cases [1-3]. The risk of strangulation and infarction has been overestimated in asymptomatic patients. However, in symptomatic patients, or if the symptoms are severe and progressive, surgery should be considered. Sometimes symptoms are intermittent and can vary from gastrointestinal complaints to dyspnea and palpitations, due to the pressure caused by the herniated stomach. It is important to refer that symptoms are often relieved with the simple passage of a nasogastric tube. Gastric infarction is a surgical emergency and therefore gastric necrosis must be excluded. Surgery is mandatory [3].

The initial evaluation of the acute gastric volvulus comprehends a plain film that classically shows a single large spherical gas bubble in the upper abdomen and a paucity of air in the distal bowel. This examination should be followed by computed tomography (CT).

Patients with chronic gastric volvulus may lack typical clinical signs and further imaging, such as upper gastrointestinal series, will be needed [2]. If there is acute gastric distention due to gastric volvulus, an immediate gastric decompression at the bedside required (with a nasogastric tube). If the latter is unsuccessful, surgical intervention or endoscopic assistance may be needed. Fluid resuscitation and correction of electrolyte abnormalities are also included in the initial management [2,3,5,6].

Urgent open abdominal exploration following resuscitation and gastric decompression is mandatory if: stomach decompression cannot be achieved by nasogastric tube or endoscopically; there is imagiological evidence of gastric perforation or mediastinal contamination; there is shock or hypotension refractory to resuscitation; severe sepsis. This urgency is linked by the high probability of necrosis and/or perforation. Surgical resection is needed for septic source control and endoscopic evaluation can assist in the diagnosis or exclusion of gastric ischemia in patients with partial clinical responses to decompression [2,3].

Patients with a chronic presentation, acute symptoms but clinically stable, and those who responded to the initial resuscitation and gastric decompression, can evade immediate surgery. These groups can undergo a later planned treatment after completing the initial diagnostic evaluation and medical/anesthetic risk assessment [2]. Appropriate treatment should be employed, in order to avoid the complications of an acute presentation such as ulceration, strangulation, necrosis and hypovolemic shock. These complications are responsible for the mortality of 30% up to 50% [7].

In the case of patients with primary symptomatic gastric volvulus (acute or chronic), gastric fixation to anchor the stomach to the abdominal wall will minimize recurrence and may be a better option than simple observation. The approach, either laparoscopic gastropexy or percutaneous endoscopic gastrostomy, will depend on many variables, such as patient characteristics and clinician preference [2,3,8]. The laparoscopic approach has become the gold standard for chronic volvulus repair.

In the case of symptomatic secondary gastric volvulus, a surgical repair of the anatomic defect is recommended in addition to gastric fixation, rather than observation or gastric fixation alone. This approach reduces the recurrence risk. Not repairing the anatomic defect is an adequate alternative, for those patients with substantial medical comorbidities, provided that gastric derotation and fixation have been accomplished [2,5,7,8].

If the medical comorbidities prohibit surgery, less invasive approaches are acceptable, such as endoscopic derotations and PEG tube placement. Multiple fixation gastrostomy has been described to prevent a recurrence, but it does not work as a definitive treatment. Some authors have reduced the stomach laparoscopically to later fix it by means of a percutaneous endoscopic gastrostomy. Others have suggested fixing the body and the stomach in the mesocolon through a mesocolic vent [5,6,8]. However, because endoscopic repair does not address anatomic abnormalities leading to the etiology, this approach is considered less optimal [2,5,8].

Evidence shows that elderly patients who undergo laparoscopic treatment of gastric volvulus associated with hiatal hernia do not usually suffer any additional perioperative morbidity, supporting that laparoscopic surgery is a safe option for these patients [5].

Conservative treatment with watchful observation seems to be a safe therapeutic option in patients with chronic gastric volvulus. The latest studies demonstrate that subjects usually do not manifest major complications during the follow-up period and only a very few decide for elective surgery. Nevertheless, symptom recurrence is a common feature and so symptoms must be addressed accordingly through pharmacological and non-pharmacological options [5,9].

## Conclusions

Gastric volvulus is a rare entity that can cause a high mortality rate in acute cases or severe morbidity with serious complications in a chronic presentation. The diagnosis is imperative although challenging due to the variable clinical presentations and difficult interpretation of the imaging studies. For those reasons, a high index of clinical suspicion is required making the presentation and discussion of case reports like the present, of high importance. Even though there are several therapeutic options including in the elderly, such as surgery, laparoscopic techniques and conservative treatment, the choice should be personalized to each patient and above all, respectful of their decision.

With an increasing average life expectancy in the world, doctors need to be prepared to manage elderly patients with multiple comorbidities, and for so, prepare to adopt less linear approaches. For these patients, the best decision may not be the complete resolution of their problem, but the minimization of symptoms and close monitoring by the health team. This case report highlighted the importance of multidisciplinary action and patient empowerment in achieving an optimized health potential through patient-centered medicine.
